# Investigation of singular and simultaneous faults in rotating machines using the finite element method

**DOI:** 10.1038/s41598-026-53898-2

**Published:** 2026-06-01

**Authors:** Natalia Espinoza-Sepulveda, Akilu Yunusa-Kaltungo

**Affiliations:** Department of Mechanical and Aerospace Engineering, Faculty of Science and Engineering, The University of Manchester, Manchester, M13 9PL UK

**Keywords:** Rotor defect, Rotor fault, Combined faults, Finite element method, Engineering, Mathematics and computing

## Abstract

Rotating machinery failures continue to impose substantial economic, operational and safety risks across critical industries such as power generation, manufacturing, oil and gas, and aerospace, where unplanned downtime can severely disrupt production and elevate maintenance costs. This study addresses a critical gap in the field of failure management by providing a comprehensive, physics‑based investigation into the dynamic behaviour of rotating machines subjected to simultaneous rotor faults. These conditions closely reflect real industrial environments yet remain insufficiently explored in the literature. A finite element (FE) model based on Timoshenko beam theory is developed, calibrated, and validated using experimental modal and vibration data from a laboratory-scale two-stage rotor rig. Five common defects -unbalance, misalignment, shaft bow, pedestal looseness, and partial rotor rub- are examined in combined scenarios to evaluate their interactions and influence on system dynamics. The validated FE model successfully reproduces the characteristic vibration signatures observed experimentally, including harmonic responses associated with unbalance and misalignment, eccentricity‑driven amplification due to shaft bow, intermittent impact behaviour from looseness, and super harmonics and chaotic components arising from rub. The study reveals insights into how coupled faults modify dynamic responses, including location‑specific amplification near natural frequencies, speed‑dependent shifts in the dominant harmonics, and impulsive responses driven by rub-bow interactions. The findings offer significant potential for advancing vibration‑based condition monitoring, providing a foundation for improved feature selection in intelligent diagnostic systems and supporting the development of robust, physics‑informed approaches for managing rotor‑related faults in industrial machinery.

## Introduction

Rotating machines are integral to the operations of vital industries such as power, oil and gas, manufacturing, construction, food processing, pharmaceuticals, and aerospace^1^. From turbines, alternators, and pumps to electric motors, multipliers, and speed reducers with gears, their essential function and criticality are evidenced by their impact on the overall performance of processes^2^. Downtime is costly for any industry, particularly when it is unexpected. Its occurrence not only signifies an economic cost, but it also has consequences for safety, product quality, potential environmental hazards, customer satisfaction, and regulatory compliance^3^.

According to Thomas and Weiss^4^ the costs of machinery failures and repairs could vary from 15% to 70% of the cost of goods produced, depending on the industry. The latest report issued by Siemens AG^5^, *“The true cost of downtime 2024”*, revealed that unplanned downtime represents 11% of annual revenues of the world’s 500 biggest companies, equating to over $1.4 trillion. Among other relevant findings, this analysis demonstrated that, when combining all examined sectors—automotive, oil and gas, heavy industries, and fast-moving consumer goods—the average cost per hour of unplanned downtime has doubled over the past five years. Heavy industry, such as steel and cement manufacturing, whose hourly cost increased by 319% in 2023 compared to 2019, has significantly impacted this average figure. The report also demonstrated that, despite increased hourly rates, the overall yearly loss from unscheduled downtime across all sectors has declined during the study period. This is consistent with the observed decrease in the frequency and duration of incidents, which is attributable to a broader adoption of preventive maintenance techniques.

According to the hierarchy of controls, total elimination of the occurrence of faults would seem the most ideal approach to industrial operations management; however, this is far from the norm in real-life settings due to random failures^3^. Failure causes can be attributed to numerous factors, including material defects, poor operation, change of intent, poor installation/commissioning, and inadequate maintenance practices. It is found that over 70% of the faults associated with rotating machinery are owed to shaft/rotor and bearing defects^6^. These faults have been the subject of extensive research, with new approaches emerging in response to technological advancements and industry needs.

In engineering, mathematical models are widely used to study the dynamic responses of rotating machines under certain operational conditions, particularly to detect the emergence of faults at their incipient stages. A great benefit of mathematical models is their ability to effectively, in terms of speed, accuracy, and costs, estimate the responses and behaviours of the studied machines and their mechanisms under low-hazard environments, since they can eliminate or significantly reduce the need for hands-on experiments or field measurements.

The finite element (FE) method is among the most used approaches to model complex rotor setups and operational conditions, such as coupled faults and variable loads. In this context, scenarios including rotor rub (both individual and combined faults) seem to get major attention in the literature.

The dynamic model of a power turbine developed by Nan, Yang and Yu^7^ was used to investigate simultaneously occurring faults, i.e. misalignment, unbalance and rub. The results obtained through the system’s equations of motion were validated using an FE model, emphasising the effects on the chaotic response of the rotor caused by changes in the stiffness of the system upon contact. Similar findings have been reported by Tofighi-Niaki, Asgharifard-Sharabiani and Ahmadian^8^, who concluded that sudden variations in impact force led to qualitative changes in the rotor response. The speed and direction of rotation have also been shown to significantly influence the stability of rotor responses under combined rub and unbalance conditions, as demonstrated through the nonlinear analysis conducted by Prabith and Krishna^9^. Building on these nonlinear dynamic investigations, Zhang, Yang^10^ introduced an energy-based diagnostic approach in which the time-averaged input power flow is employed to characterise rub-impact severity. Their analysis showed that rubbing introduces additional contact and friction forces that alter the system’s energy exchange, leading to a notable reduction in the time-averaged power flow as severity increases, which was experimentally validated and demonstrated on practical rotor systems.

Numerous studies have been conducted about blade-casing rub in aeroengines. For example, Chen^11^ incorporated geometrical parameters into their model due to the relative rotor-stator position and number of blades. This model was designed to assess different rubbing conditions, and its generated responses were validated via an experimental setup. Hong, Li^12^ developed a FE model of an aeroengine experimental rig to investigate structural effects in the blades, including deformation and stress. The authors advocated the usefulness of the model for design quantification analysis, owing to the consistency of the experimental and numerical results.

Using a multi-bearing FE model, Wang, Liu and Jiang^13^ investigated how to predict the transient dynamic response of a dual rotor blade casing system with the blade off in a turbofan engine, and realised that asymmetry due to the missing blade initiates rub, while abrupt unbalance results in impact loads. Consequently, the study demonstrates how inertia asymmetry significantly affects the machine’s transient responses. Similarly, Zeng, Ma^14^ studied rub in a single-blade-casing system with flexible supports, using a two-node Timoshenko beam FE model and implementing two contact interface techniques. While the study is presented only in terms of numerical simulation, it shows important findings regarding the effects of increased friction in the system on the non-linear behaviour in the presence of rub, shifting the local resonance frequency.

Mokhtar, Darpe and Gupta^15^ also developed a model to study rub via a coupled rotor-stator system, aiming to provide insights about the actual dynamic interaction between the components that are coupled through bearing supports. While most of the existing studies on contact or rub in rotors focus on rotor-stator interaction, Ma, Li^16^ used Timoshenko beam theory and the FE method to investigate disc-shaft contact in the presence of parallel misalignment. The measured dynamic responses indicated the presence of shaft-disc impact at particular speeds and levels of misalignment, as the friction and clearance between the two components varied.

Xiang, Deng^17^, Xiang, Gao and Hu^18^, Xiang, Zhang and Hu^19^ investigated coupled faults involving crack, rub-impact and oil-film instability, whereby a simplified mathematical model consisting of shaft, disc and two bearings as supports was proposed, while neglecting the effects of shear, torsional vibration and gyroscopic coupling. It was found that stator stiffness and crack depth affect rotor stability. Furthermore, substantial nonlinearity was observed at high speeds, which is consistent with experimental findings.

Although rotor rub and other nonlinear faults, such as shaft cracks^20^, have been widely examined in their combined forms, the literature indicates that bent shaft behaviour remains comparatively underexplored, most often addressed only when it co-occurs with unbalance. Chen^21^ demonstrated that residual coupled bow–unbalance conditions significantly influence rotor dynamics, amplifying lateral vibration responses, altering phase and contact characteristics in geared rotor systems, and modifying critical speeds. Moreover, experimental and numerical evidence showed that bow and unbalance generate distinct amplitude–phase signatures, enabling their simultaneous identification through reduced order modelling and correlation analysis techniques^22^. Complementing this evidence, other researchers have focused on differentiating bow-induced effects from unbalance forces, emphasising the speed-independent nature of bent shaft responses relative to the speed-dependent behaviour characteristic of unbalance^20^.

**Table 1:** Summary of studies on combined rotor faults using numerical simulation.

Study	Considered faults	Research contribution	Methodology	Limitations
Nan, Yang and Yu^7^ (2024)	Misalignment + rub + unbalance	Showed that rubbing increases effective stiffness and raises critical speed; rub‑induced stiffness promoted chaotic behaviour and instability; larger clearance mitigated rub.	Lagrangian nonlinear model validated using FE analysis.	Single‑disk simplification; gyroscopic effects and shaft mass neglected; numerical only; no experimental validation.
Tofighi-Niaki, Asgharifard-Sharabiani and Ahmadian^8^ (2018)	Rub + unbalance	Identified sudden changes in rub‑impact forces causing qualitative changes in rotor response, including chaotic motion at higher speeds.	Nonlinear mathematical model incorporating dynamics and lubrication theory.	Two‑bearing rotor; structural damping neglected; numerical only; no experimental validation.
Prabith and Krishna^9^ (2021)	Rub + unbalance	Detailed stability & bifurcation mapping; shows effect of rotation direction on rotor’s critical speed, as well as dependency of whirling direction on rotational speed.	1-D FE model	Focuses on annular rub; computationally heavy.
Zhang, Yang^10^ (2023)	Rub + unbalance	Introduces a time‑averaged input power‑flow indicator; links rubbing severity with energy dissipation.	FE modelling; experimental validation.	Experiments at low speed; mainly single‑point rubbing.
Chen^11^ (2016)	Rub + unbalance	Developed an elastic rubbing model embedded within a rotor–support–casing FE system.	FE model with laboratory validation for selected rubbing patterns.	Two‑bearing rotor; misalignment not included; experimental envelope limited.
Hong, Li^12^ (2019)	Rub + unbalance	Observed a transition from synchronous‑dominant response to strong super-harmonics as rub severity increased; lower yield strength or Young’s modulus increased deformation, contact time and stress concentration.	2‑D/3‑D FE modelling with experimental validation.	Two‑bearing rotor; misalignment not included; damping representation introduces quantitative limits; single operating speed studied.
Wang, Liu and Jiang^13^ (2019)	Rub + unbalance + misalignment	Found that rubbing constrains motion and reduces vibration peaks; identified parametric instability under high inertia asymmetry; analysed transient blade‑off response.	Dual‑rotor FE model.	Numerical only; no experimental validation.
Zeng, Ma^14^ (2019)	Rub + unbalance	Showed severe rubbing produces resonance bands and sidebands; higher friction induces soft nonlinearity; strain‑energy and spectral metrics identify dominant modes.	FE model.	Numerical only; no experimental validation; single‑blade simplification.
Mokhtar, Darpe and Gupta^15^ (2018)	Rub + unbalance	Proposed stator vibration alone can diagnose rubbing; transient rub excitation increases natural frequencies.	FE model.	Two‑bearing rotor–stator; numerical only; no misalignment; sensitive to assumed damping ratio.
Ma, Li^16^ (2025)	Rub (disc–shaft) + misalignment + unbalance	Demonstrated that nonlinear disc–shaft contact generates jagged orbits and superharmonics; misalignment amplifies collision severity.	FE Timoshenko beam rotor model with experimental validation.	Two‑bearing single‑disk rotor; sensitive to assumed damping and support stiffness/damping.
Chen^21^ (2019)	Bow + unbalance	Showed residual bow substantially increases lateral response amplitudes and alters gear‑mesh behaviour.	FE Timoshenko beam rotor with gears and isotropic bearings.	Numerical only; no experiments; sensitive to assumed damping and stiffness parameters.
Juethner, Rose^23^ (2023)	Bow + unbalance	Found speed‑independent synchronous forcing from bow dominates response, while geometric grid deviations are secondary.	1‑D beam FE model with disks.	Simulated case studies; no misalignment; sensitive to bearing parameters.
Sanches and Pederiva^22^ (2016)	Shaft bow + unbalance	Developed correlation‑analysis with model‑order reduction to identify bow and unbalance magnitudes and phases.	FE rotor model with experimental validation.	Two‑bearing single‑disc rotor; misalignment not included; assumes synchronous faults.

Accurately predicting the dynamic response of rotating machinery requires an in-depth understanding of how multiple faults interact, particularly when nonlinear mechanisms such as looseness and rotor rub are present. Although individual defects have been extensively studied, real machines often operate under several concurrent faults whose combined effects remain challenging to diagnose and model. The intrinsic complexity of these systems, together with the strong nonlinearities associated with common defects, continues to limit the reliability of traditional fault diagnosis techniques. Nonetheless, modelling a system whose responses are sufficiently similar to those of the experiments is a valuable engineering tool that can support further developments and decisions within industrial environments. Despite the significance of the knowledge generated by previous studies on rotating machines’ fault diagnosis, especially regarding the signatures of different faults, very few studies have extensively examined multiple combined scenarios using physics-based approaches (Table 1), which is particularly true for bent shaft cases. The limited instances of such include those demonstrated in earlier studies^24–30^, which have explored fault identification using experimentally acquired data across varying speeds, fault types, and support flexibilities, largely relying on data fusion approaches. As a result, they offer limited theoretical insight into whether the observed behaviours arise from consistent and repeatable physical mechanisms.

Motivated by these limitations, the present work proposes a comprehensive physics-based investigation into combined rotor faults using a finite element modelling framework calibrated and validated against experimental measurements. First, modal test data are employed to update key dynamic parameters, such as bearing–pedestal stiffness and global system damping, ensuring that the numerical model reflects the true characteristics of the machine. Subsequently, measured responses across multiple operating speeds and individual fault conditions are used to validate the capability of the model, with the different defects introduced by integrating several existing modelling approaches. Finally, the validated single fault models are used to construct multi-fault scenarios, enabling systematic exploration of how interacting defects influence the overall dynamic response. This approach provides new insight into the dominant mechanisms governing complex fault interactions and advances the development of predictive, physics-grounded diagnostics for multi-fault rotor systems.

## Studied rotor

A numerical simulation of a laboratory-scale rotor-bearing system was developed using the FE method. The experimental rig was extensively used to understand the dynamic characteristics of rotating machines under different rotor-related faults in earlier studies^24–30^. To validate the experimental data-based findings reported in their previous studies^31,32^, Espinoza-Sepúlveda and Sinha^33–35^ used data sets generated in a FE model representation of the rig. While the current study is based on a similar approach, it considers other fault scenarios, including individual and combined defects, as well as an updated FE model to improve the representation of the studied machine’s dynamic responses.

### Experimental setup and data

**Fig. 1:**
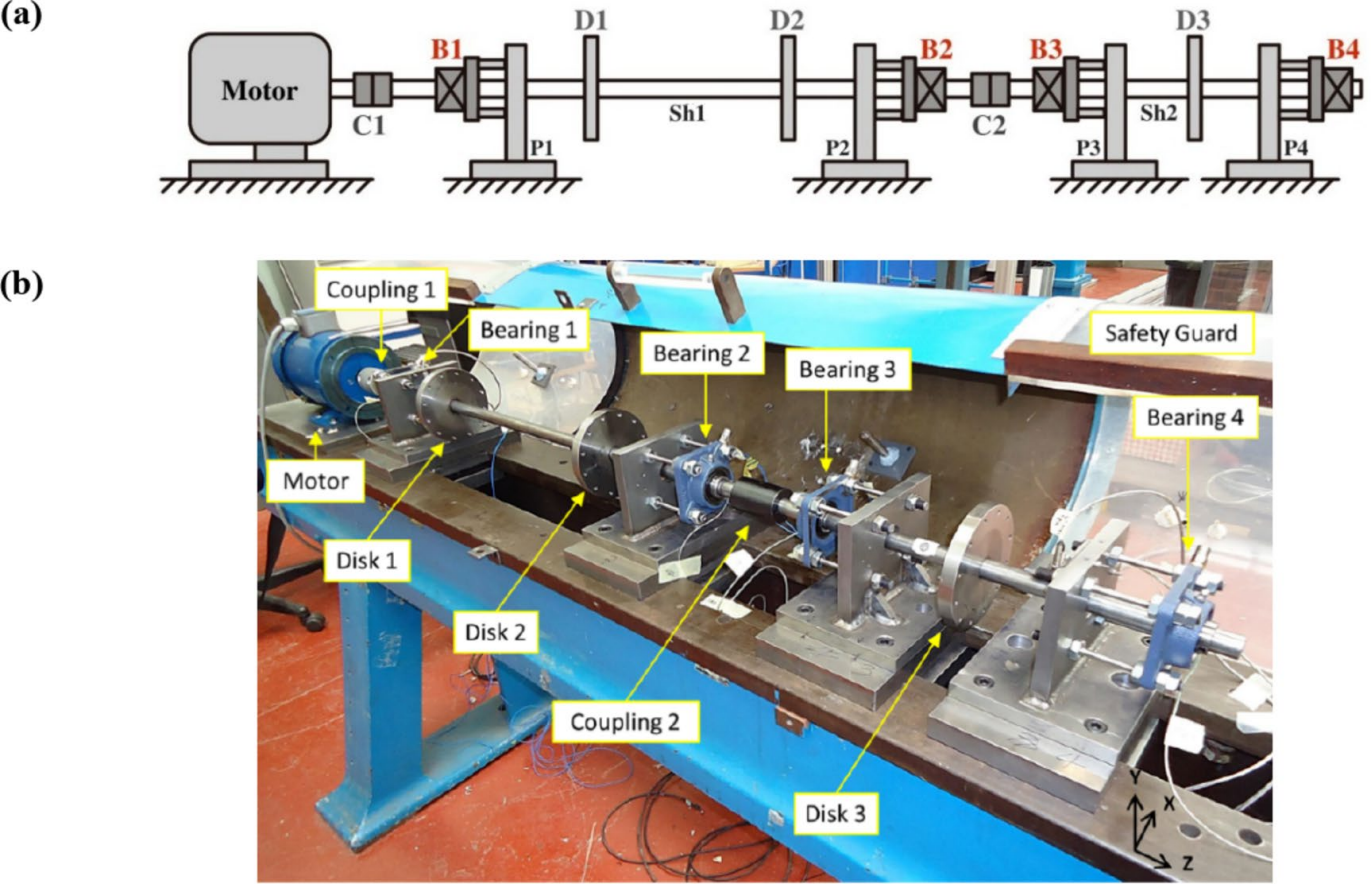
(**a**) Schematic representation of the studied machine^35^. (**b**) Experimental setup^27^.

As depicted in Fig. 1(a), the studied machine represents a typical two-stage rotor that comprises two mild steel shafts, Sh1 and Sh2, connected through a rigid coupling (C2). Sh1 and Sh2 have lengths of * 1000mm* and * 500mm*, respectively, both with a nominal diameter of 20 mm.

The entire assembly is mounted on four identical bearings (B1 to B4) and pedestals (P1 to P4). A * 3000rpm* electric motor is flexibly coupled (C1) to the rotor-bearing assembly via the end of Sh1, supported by B1 and P1.

Additionally, three identical balancing discs are distributed along the shafts, i.e. D1, D2 mounted on Sh1, and D3 mounted on Sh2.

Further specifications of the remaining rig components are provided in Table 2.

**Table 2:** Motor, bearings and couplings general information.

Rig Component	Name on schematic	Make	Model	Description
Electric motor	Motor	Crompton Greaves	GF7965	⋅ 3 phase ⋅ 70 kW ⋅ 3000 rpm
Anti-friction ball bearing	B1, B2, B3, B4	SKF	FY20TF/RCJY20/ SF20	⋅ Flange mounted ⋅ Grease lubricated ⋅ 20 mm internal diameter ⋅ 47 mm external diameter
Flexible coupling	C1	Ruland	FCMR38-16-16-A	⋅ 16 mm x 20 mm bore
Rigid coupling	C2	n/a	n/a	⋅ 20 mm x 20 mm bore

Existing experimental data collected from earlier rotor fault studies at The University of Manchester are used to update the FE model developed in this current work.

In the laboratory setup shown in Fig. 1(b), accelerometers, powered by signal conditioning units, were used to collect the vibration signals from the machine under different operational conditions. A total of four accelerometers were utilised, one per bearing location, mounted at 45 degrees on the edge of the bearing cases. Vibration signals were simultaneously collected from the four measuring locations and stored using a customised LABVIEW-based data acquisition software designed by Austin consultants for The University of Manchester. Samples were collected during machine operation at steady speeds of * 1800rpm* or * 30Hz* and * 2400rpm* or * 40Hz*, using a sampling frequency of $\:10\:kHz$.

The initial condition, subject to residual unbalance and residual misalignment, is considered the baseline for this study. Additionally, data sets corresponding to four common rotor faults were obtained at both operational speeds. The experimentally simulated defects include misalignment, shaft bow, pedestal looseness, and partial rub, with their detailed descriptions provided in Table 3^27^.

**Table 3:** Description of experimentally simulated rotor conditions.

Rotor condition	Location	Description
Baseline (residual misalignment and residual unbalance)	n/a	Considered as the baseline condition without external faults introduced.
Misalignment	P1	1 shim at $\:0.8\:mm$, vertical
Bow	Sh1	$\:3.2\:mm$ maximum deflection, introduced at centre of shaft 1.
Looseness	P3	The four nuts at P3 are loosened.
Rub	Near D1	Simulated with an acrylic rectangular disc positioned with a gap of $\:0.1\:mm$ from the shaft surface.

In addition to the operational samples, data related to the modal test conducted on the laboratory rig are considered to estimate relevant dynamic characteristics for the FE model, such as proportional damping, support stiffness, and corroborate natural frequencies and their respective mode shapes.

### FE model approach

The rig is analysed by a shaft-line model. The structure is divided into $\:n=150\:$Timoshenko beam elements of a length * le=10mm*, with a total of $\:\left(n+1\right)=151$ nodes. The components of the rotor (i.e. couplings, bearings and balancing discs) are represented by their dynamic characteristics at their respective nodes. Since one of the core aspects of this study involves investigating lateral vibrations, four generalised coordinates are defined at each *i-node*, i.e. $\:{u}_{i}$, $\:{v}_{i}$, $\:{\theta\:}_{i}$, $\:{\psi\:}_{i}$, Thus, the vector of generalised coordinates of the element is given by $\:{q}_{e}={\left\{{u}_{i}\:{v}_{i}\:{\theta\:}_{i}\:{\psi\:}_{i}\:{u}_{i+1}\:{v}_{i+1}\:{\theta\:}_{i+1}\:{\psi\:}_{i+1}\}\right.}^{T}$.

**Fig. 2:**
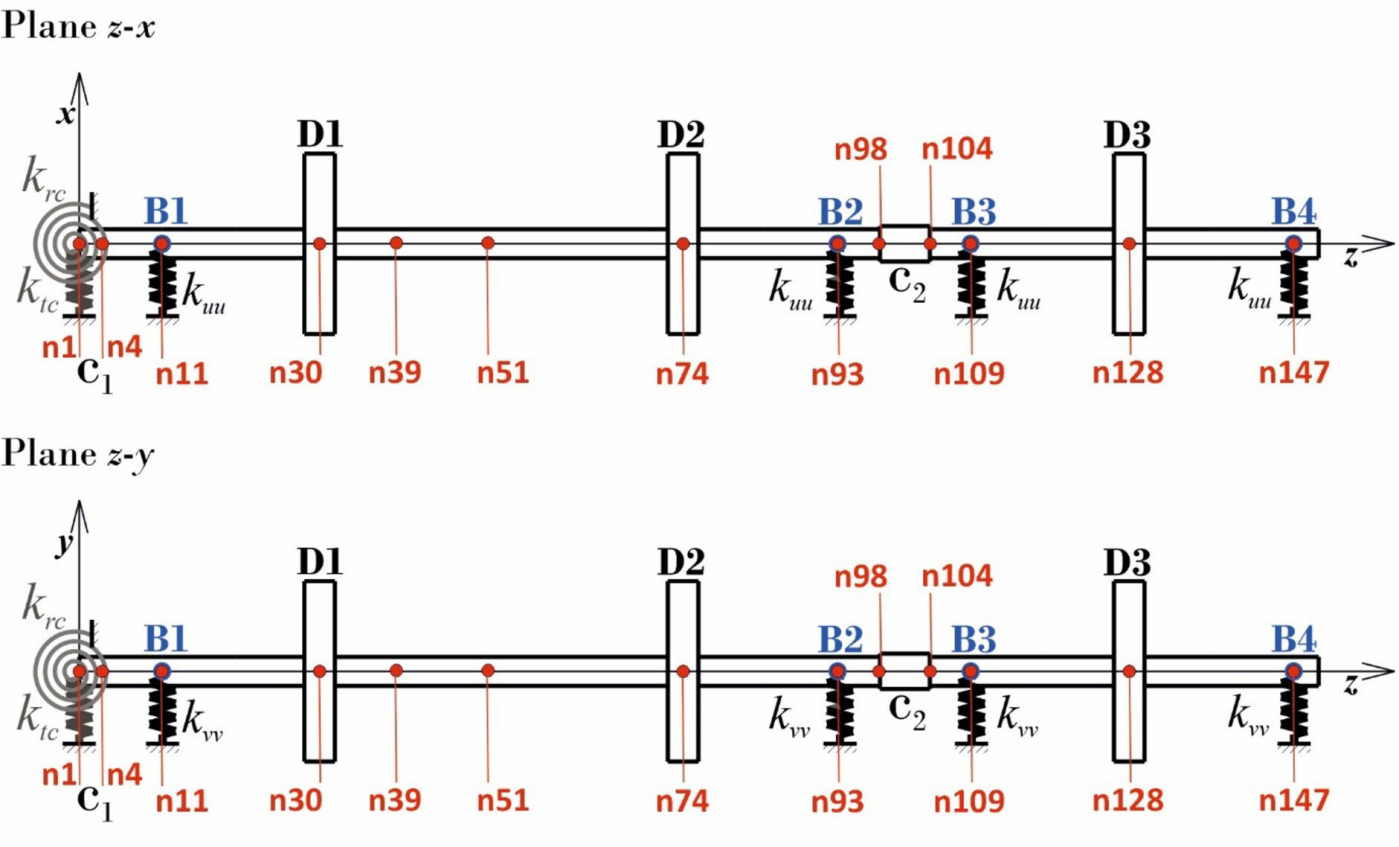
Rotor discretisation and location of components.

The rotor components are localised as seen in Fig. 2, while Table 4 presents the model elements and their respective node locations.

**Table 4:** Rotor components represented in Fig. 3 and their node locations.

Rotor component	Abbreviation	Node location
Long shaft	Sh1	1–98
Short shaft	Sh2	104–151
Flexible coupling, driven end of shaft	C1	1–4
Rigid coupling between shafts	C2	98–104
Balancing discs on longer shaft	D1; D2	30; 74
Balancing disc on shorter shaft	D3	128
Bearings supporting longer shaft	B1; B2	11; 93
Bearings supporting shorter shaft	B3; B4	109; 147

Sh1, Sh2 and C2 are modelled through Timoshenko beam elements^36^, all with a material density of $\:\mathrm{7,800}kg/{m}^{3}$. The radius of both shaft elements is * 10mm*, while the coupling has a radius of * 23mm*.

The Timoshenko beam theory includes in its calculations the effects of shear and rotary inertia, which is reflected through the shape functions in the solution of the energy equations. Therefore, for this study, the element stiffness matrix $\:{\boldsymbol{K}}_{\boldsymbol{e}}$ is obtained from the strain energy; the mass matrix for the beam elements, $\:{\boldsymbol{M}}_{\boldsymbol{e}}$, is obtained from the kinetic energy, while the gyroscopic matrix, $\:{\boldsymbol{G}}_{\boldsymbol{e}}$, is determined from the increment in kinetic energy in the shaft element^37^ and it includes the shear effects.

Since there are no physical parameters defined for damping as per mass and stiffness, a simplified model of proportional damping is implemented. The damping matrix $\:\left[\boldsymbol{C}\right]$ is considered to be proportional to $\:\left[\boldsymbol{M}\right]$ and $\:\left[\boldsymbol{K}\right]$, as shown in Eq. 1, where * a* and * b* are positive real constants.

1 $$\:\left[\boldsymbol{C}\right]=a\left[\boldsymbol{M}\right]+b\left[\boldsymbol{K}\right]$$

The damping ratios $\:{\zeta\:}_{n1}$ and $\:{\zeta\:}_{n2}$ are estimated from the experimental modal test data in the vertical direction, using half power point (HPP) method and two natural frequencies from the FRF plot in the vertical direction^27^, namely $\:{f}_{ny1}=\:50.66\:Hz$ and $\:{f}_{ny2}=59.20\:Hz$. The values for * a* and * b* are calculated, resulting in * a=0.604385102934616* and $\:b=9.142147236893553\times\:{10}^{-5}$.

Therefore, the damping force $\:{\boldsymbol{Q}}_{\boldsymbol{d}\boldsymbol{a}\boldsymbol{m}\boldsymbol{p}\boldsymbol{i}\boldsymbol{n}\boldsymbol{g}}\:$is given by Eq. 2, with * a,b* scalar constants and $\:{\Omega\:}$ the rotational speed. While the first and second terms in Eq. 8 are proportional to $\:{\boldsymbol{M}}_{\boldsymbol{e}}$ and $\:{\boldsymbol{K}}_{\boldsymbol{e}}$, respectively, the matrix $\:{\boldsymbol{K}}_{\boldsymbol{i}\boldsymbol{D}\boldsymbol{e}}$ (Eq. 3) represents the skew-symmetric contribution of internal damping to the stiffness matrix, which is linearly proportional to $\:{\Omega\:}$ and independent from oscillation frequency^37^.

2 $$\:{\boldsymbol{Q}}_{\boldsymbol{d}\boldsymbol{a}\boldsymbol{m}\boldsymbol{p}\boldsymbol{i}\boldsymbol{n}\boldsymbol{g}}=-a{\boldsymbol{M}}_{\boldsymbol{e}}{\ddot{q}}_{e}-b{\boldsymbol{K}}_{\boldsymbol{e}}{\dot{q}}_{e}-b\varOmega\:{\boldsymbol{K}}_{\boldsymbol{i}\boldsymbol{D}\boldsymbol{e}}{q}_{e}$$

3 $$\:{\boldsymbol{K}}_{\boldsymbol{i}\boldsymbol{D}\boldsymbol{e}}=\frac{EI}{\left(1+\varPhi\:\right){{l}_{e}}^{3}}\left[\begin{array}{cccccccc}0&\:12&\:-6{l}_{e}&\:0&\:0&\:-12&\:-6{l}_{e}&\:0\\\:-12&\:0&\:0&\:-6{l}_{e}&\:12&\:0&\:0&\:-6{l}_{e}\\\:6{l}_{e}&\:0&\:0&\:\left(4+\varPhi\:\right){{l}_{e}}^{2}&\:-6{l}_{e}&\:0&\:0&\:\left(2-\varPhi\:\right){{l}_{e}}^{2}\\\:0&\:6{l}_{e}&\:-\left(4+\varPhi\:\right){{l}_{e}}^{2}&\:0&\:0&\:-6{l}_{e}&\:-\left(2-\varPhi\:\right){{l}_{e}}^{2}&\:0\\\:0&\:-12&\:6{l}_{e}&\:0&\:0&\:12&\:6{l}_{e}&\:0\\\:12&\:0&\:0&\:6{l}_{e}&\:-12&\:0&\:0&\:6{l}_{e}\\\:6{l}_{e}&\:0&\:0&\:\left(2-\varPhi\:\right){{l}_{e}}^{2}&\:-6{l}_{e}&\:0&\:0&\:\left(4+\varPhi\:\right){{l}_{e}}^{2}\\\:0&\:6{l}_{e}&\:-\left(2-\varPhi\:\right){{l}_{e}}^{2}&\:0&\:0&\:-6{l}_{e}&\:-\left(4+{l}_{e}\right){{l}_{e}}^{2}&\:0\end{array}\right]$$

C1 is located between nodes 1 and 4 (n1 and n4), at the inboard end of the rotor. This a flexible coupling which is modelled by adding its mass and stiffness to the corresponding nodes and neglecting inertia values. The total coupling mass, $\:{m}_{c1}=0.2359\:kg$ was added to the model in half and distributed over n1 to n4. The stiffness matrix for the flexible coupling in Eq. 4, $\:{\boldsymbol{K}}_{\boldsymbol{c}1}$, has been determined following the approach by Saavedra and Ramírez^38^. The translational and rotational stiffness values, given by the manufacturer, are $\:{k}_{tc}=27\:kN/m$ and $\:{k}_{rc}=25\:Nm/rad$, respectively.

4 $$\:{\boldsymbol{K}}_{\boldsymbol{c}1}=\left[\begin{array}{cccccccc}{k}_{tc}&\:0&\:0&\:\frac{{k}_{tc}*{l}_{c}}{2}&\:-{k}_{tc}&\:0&\:0&\:\frac{{k}_{tc}*{l}_{c}}{2}\\\:0&\:{k}_{tc}&\:-\frac{{k}_{tc}*{l}_{c}}{2}&\:0&\:0&\:-{k}_{tc}&\:-\frac{{k}_{tc}*{l}_{c}}{2}&\:0\\\:0&\:-\frac{{k}_{tc}*{l}_{c}}{2}&\:\frac{{k}_{tc}*{{l}_{c}}^{2}}{4}+{k}_{rc}&\:0&\:0&\:\frac{{k}_{tc}*{l}_{c}}{2}&\:\frac{{k}_{tc}*{{l}_{c}}^{2}}{4}-{k}_{rc}&\:0\\\:\frac{{k}_{tc}*{l}_{c}}{2}&\:0&\:0&\:\frac{{k}_{tc}*{{l}_{c}}^{2}}{4}+{k}_{rc}&\:-\frac{{k}_{tc}*{l}_{c}}{2}&\:0&\:0&\:\frac{{k}_{tc}*{{l}_{c}}^{2}}{4}-{k}_{rc}\\\:{-k}_{tc}&\:0&\:0&\:-\frac{{k}_{tc}*{l}_{c}}{2}&\:{k}_{tc}&\:0&\:0&\:-\frac{{k}_{tc}*{l}_{c}}{2}\\\:0&\:-{k}_{tc}&\:\frac{{k}_{tc}*{l}_{c}}{2}&\:0&\:0&\:{k}_{tc}&\:-\frac{{k}_{tc}*{l}_{c}}{2}&\:0\\\:0&\:-\frac{{k}_{tc}*{l}_{c}}{2}&\:\frac{{k}_{tc}*{{l}_{c}}^{2}}{4}-{k}_{rc}&\:0&\:0&\:\frac{{k}_{tc}*{l}_{c}}{2}&\:\frac{{k}_{tc}*{{l}_{c}}^{2}}{4}+{k}_{rc}&\:0\\\:\frac{{k}_{tc}*{l}_{c}}{2}&\:0&\:0&\:\frac{{k}_{tc}*{{l}_{c}}^{2}}{4}-{k}_{rc}&\:-\frac{{k}_{tc}*{l}_{c}}{2}&\:0&\:0&\:\frac{{k}_{tc}*{{l}_{c}}^{2}}{4}+{k}_{rc}\end{array}\right]$$

The three discs in the rig are identical and modelled as rigid discs at one node each (n30, n74 and n128), represented by their mass and gyroscopic matrices. The outer and inner diameters are $\:{D}_{o}=125\:mm$ and $\:{D}_{i}=20\:mm$ respectively; thickness is $\:{h}_{d}=14\:mm$, and density is considered to be the same as the shaft ($\:7800\:kg/{m}^{3}$).

Bearings are modelled as concentrated masses at the corresponding nodes. All four bearing units are the same, with a total mass per unit $\:{m}_{b}=0.54kg$.

Values of bearing stiffness themselves are considerably high; thus, determining its impact on the model will be a function of the supports and foundation values. The assembly is mounted on four identical supports, which are modelled as linear springs. Their contribution to the stiffness matrix in their respective nodes is given by $\:{k}_{uu}$ and $\:{k}_{vv}$. The obtained stiffnesses in horizontal and vertical directions are given by $\:{k}_{uu}=\mathrm{16,912,000}\:N/m\:$and $\:{k}_{vv}=\mathrm{2,590,000}\:N/m$, respectively. These values are determined by iterations in the FE model, aiming to match the first two natural frequencies of the rotor.

The used natural frequencies and corresponding mode shapes were obtained from earlier modal tests conducted by Nembhard, Sinha and Yunusa-Kaltungo^27^. In their study, impacts were applied at two locations, positioned between B1–D1 and B3–D3, respectively. For each location, ten repeated impacts were recorded in both the vertical and horizontal radial directions across nine measurement points. The resulting data were then processed to compute the Frequency Response Functions (FRFs) and extract the mode shapes.

The equation of motion represents the complete integration of the entire system. Just to remark on the consideration of gyroscopic effect and internal damping contribution to stiffness, both matrices dependent on rotational speed Ω have been written separately from the general stiffness and damping matrices in Eq. 5. For convenience, in future calculations, internal damping contribution to the stiffness matrix, $\:\mathrm{B}{\Omega\:}{\boldsymbol{K}}_{\boldsymbol{i}\boldsymbol{D}}$, could form part of $\:\boldsymbol{K}$ matrix. The forces vector, $\:\boldsymbol{F}$, contains the external forces applied to the rotor.

5 $$\:\boldsymbol{M}\ddot{\boldsymbol{q}}+\left(\boldsymbol{C}+\varOmega\:\boldsymbol{G}\right)\dot{\boldsymbol{q}}+\left(\boldsymbol{K}+b\varOmega\:{\boldsymbol{K}}_{\boldsymbol{i}\boldsymbol{D}}\right)\boldsymbol{q}=\boldsymbol{F}$$

### Natural frequencies

The errors of the numerically estimated natural frequencies with respect to the experimental results^27^ are listed in Table 5. Despite the low error obtained in the first 2 natural frequencies, it is important to note that the remaining natural frequencies and mode shapes were neither studied nor considered during the iteration process of stiffness adjustment for the supports.

**Table 5:** Experimental and numerically simulated natural frequencies of the studied rig.

Natural Frequency	Experimental (Hz)	Mathematical (Hz)	Error (%)	Dominant Direction
$\:{f}_{n1}$	50.6600	50.6638	$\:+7.4840\times\:{10}^{-3}$	Y-direction
$\:{f}_{n2}$	56.7600	56.7649	$\:+8.6236\times\:{10}^{-3}$	X-direction

## Rotor defects and system response estimation

Five typical rotor-related faults have been considered in this study, i.e. unbalance, misalignment, bent shaft, rotor rub and mechanical looseness in the pedestal.

The system responses are estimated through the Newmark integration method^39^. The solution of the equation of motion is obtained assuming a constant acceleration between the times $\:{t}_{i}$ and $\:({t}_{i}+\varDelta\:t)$ of the simulation, with coefficients $\:{\alpha\:}_{N}=1/4$ and $\:{\beta\:}_{N}=1/2$ for the stability of the system. The dynamic characteristics in the equations of motion will vary according to the specific rotor condition simulated.

All the rotor-related conditions were simulated under steady speed, namely * 1800rpm* or $\:30\:Hz$ and * 2400rpm* or * 40Hz*. The vibration signals were generated to match a sampling frequency $\:{f}_{s}={10}^{4}\:Hz$, which defines a $\:\varDelta\:t={10}^{-4}s$. Finally, random noise with a signal-to-noise ratio (SNR) of $\:25\:dB$ was added to the generated responses.

### Unbalance

Unbalance forces, $\:{F}_{u}$, arise in the system when the mass centreline differs from the equilibrium position during operation of the machine.

Defining the difference in centrelines position through the rotor as $\:{\boldsymbol{q}}_{\boldsymbol{\epsilon\:}}$, and replacing $\:\boldsymbol{q}$ in the system’s equation of motion by $\:(\boldsymbol{q}+{\boldsymbol{q}}_{\boldsymbol{\epsilon\:}})$ and $\:{\ddot{\boldsymbol{q}}}_{\boldsymbol{\epsilon\:}}=-{{\Omega\:}}^{2}{\boldsymbol{q}}_{\boldsymbol{\epsilon\:}}$, results into Eq. 6.

6 $$\:\mathbf{M}\ddot{\boldsymbol{q}}+{\Omega\:}\mathbf{G}\dot{\boldsymbol{q}}+\mathbf{C}\dot{\boldsymbol{q}}+\boldsymbol{K}\boldsymbol{q}={{\Omega\:}}^{2}\mathbf{M}{\boldsymbol{q}}_{\boldsymbol{\epsilon\:}}-{\Omega\:}\mathbf{G}{\dot{\boldsymbol{q}}}_{\boldsymbol{\epsilon\:}}$$

For a particular node * i*, writing the displacement and velocity in the complex form, Eq. 7 is obtained for $\:{F}_{u}$, where $\:\mathfrak{K}\left(\:\right)\:$is the real part of $\:\left(\:\right)$, $\:{{\Omega\:}}^{2}{b}_{0e}$ is the out-of-balance forces vector acting over the node * i* with the frequency of the rotor rotation $\:{\Omega\:}$ with an offset angle $\:\beta\:$. The angles of the unbalance force and moment with respect to the * Oxy* axes are given by $\:\delta\:$ and $\:\gamma\:$ are^37^.

7 $$\:{{\Omega\:}}^{2}{\boldsymbol{M}}_{\boldsymbol{i}}{\boldsymbol{q}}_{\boldsymbol{\epsilon\:}}-{\Omega\:}{\boldsymbol{G}}_{\boldsymbol{i}}\dot{{\boldsymbol{q}}_{\boldsymbol{\epsilon\:}}}=\mathfrak{K}\left({{\Omega\:}}^{2}\left\{\begin{array}{c}{m}_{i}\epsilon\:{e}^{j\delta\:}\\\:-j{m}_{i}\epsilon\:{e}^{j\delta\:}\\\:j\left({I}_{di}-{I}_{pi}\right)\beta\:{e}^{j\gamma\:}\\\:\beta\:\left({I}_{di}-{I}_{pi}\right){e}^{j\gamma\:}\end{array}\right\}{e}^{j{\Omega\:}t}\right)=\mathfrak{K}\left({{\Omega\:}}^{2}{\boldsymbol{b}}_{0\boldsymbol{e}}{e}^{j{\Omega\:}t}\right)={F}_{u}$$

### Misalignment

The vector of the misalignment force $\:{F}_{m}$ is calculated as Eq. 8, using the approach described by Saavedra and Ramírez^38^, where $\:{\boldsymbol{K}}_{\boldsymbol{L}\boldsymbol{c}1}$ and $\:{\boldsymbol{K}}_{\boldsymbol{U}\boldsymbol{c}1}$ are the lower and upper halves of the flexible coupling’s stiffness matrix, $\:{\boldsymbol{K}}_{\boldsymbol{c}1}$ in Eq. 4.

8 $$\:{\boldsymbol{F}}_{\boldsymbol{m}}=\left[{\boldsymbol{K}}_{\boldsymbol{L}\boldsymbol{c}1}\right]*\left\{\begin{array}{c}\varDelta\:x\\\:\varDelta\:y\\\:{\theta\:}_{x}\\\:{\theta\:}_{y}\\\:0\\\:0\\\:0\\\:0\end{array}\right\}-{[\boldsymbol{K}}_{\boldsymbol{U}\boldsymbol{c}1}]*\left\{\begin{array}{c}0\\\:0\\\:0\\\:0\\\:\varDelta\:x\\\:\varDelta\:y\\\:{\theta\:}_{x}\\\:{\theta\:}_{y}\end{array}\right\}$$

where $\:\varDelta\:x$ and $\:\varDelta\:y$ are the misaligned positions in horizontal and vertical directions with respect to the centreline of the flexible coupling without deformation, while $\:{\theta\:}_{m,x}$ and $\:{\theta\:}_{m,y}$ are the misalignment angles. The force $\:{F}_{m}$ is applied to node 4, n4, which corresponds to the end position of the coupling on the electric rotor side, acting at $\:{\Omega\:}$ and $\:2\times\:{\Omega\:}$ frequencies.

### Bent shaft

**Fig. 3:**
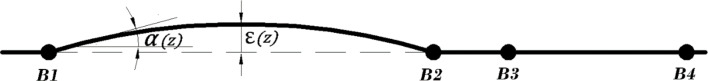
Deflection of Sh1 in the shaft bow simulation.

The simulation of bent shaft was carried out by considering a known deflection of Sh1, between B1 and B2, which has a parabolic form as described in Fig. 3. The forces are calculated by considering a variable eccentricity along the shaft, which is determined as a function of the axial position * z*, with a maximum deformation of $\:3.2\:mm$ at its centre.

Defining the vector $\:{\boldsymbol{q}}_{\boldsymbol{E}}$ as the elastic deflections and $\:{\boldsymbol{q}}_{\boldsymbol{B}}$ as the static deflections regarding the bent shaft, the total deflections at the nodes, $\:\boldsymbol{q}$, are given by $\:\boldsymbol{q}={\boldsymbol{q}}_{\boldsymbol{E}}+{\boldsymbol{q}}_{\boldsymbol{B}}$. Thus, the equation of motion results into Eq. 9.

9 $$\:\boldsymbol{M}\ddot{\boldsymbol{q}}+\left({\Omega\:}\boldsymbol{G}+\boldsymbol{C}\right)\dot{\boldsymbol{q}}+\boldsymbol{K}\boldsymbol{q}=\boldsymbol{K}{\boldsymbol{q}}_{\boldsymbol{B}}$$

Because of the rotation of the shaft, $\:{\boldsymbol{q}}_{\boldsymbol{B}}$ is a function of time and at the $\:i\:$node is given by $\:{q}_{Bi}\left(t\right)=\:{\mathfrak{K}(q}_{B0i}{e}^{j{\Omega\:}t})$, where $\:{q}_{B0i}$ is determined as per the unbalance simulation, evaluating the respective eccentricity $\:\epsilon\:\left(z\right)$ and slope $\:\alpha\:\left(z\right)$ (Eq. 10). If the rotor position shown in Fig. 3 is considered the starting position, the angle of the force is $\:\delta\:=\pi\:/2\:rad$ and the angle of the moment $\:\gamma\:=0$.

10 $$\:{\boldsymbol{q}}_{\boldsymbol{B}0\boldsymbol{i}}=\mathfrak{K}\left(\left\{\begin{array}{c}\epsilon\:{e}^{j\delta\:}\\\:-j\epsilon\:{e}^{j\delta\:}\\\:j\alpha\:{e}^{j\gamma\:}\\\:\alpha\:{e}^{j\gamma\:}\end{array}\right\}{e}^{j{\Omega\:}t}\right)$$

### Mechanical looseness in the pedestal

**Fig. 4:**
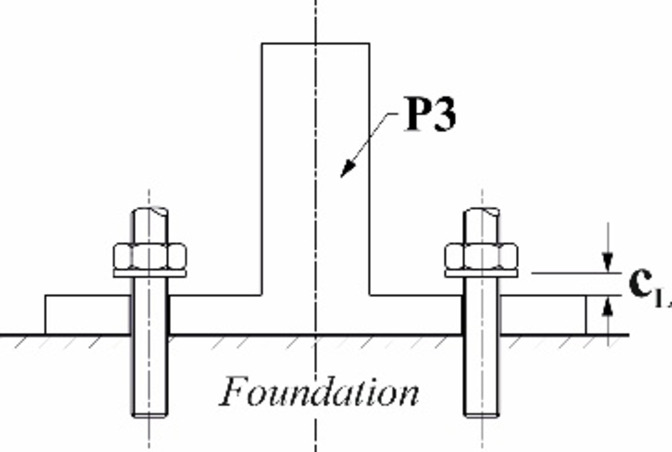
Looseness in bearing pedestal.

Mechanical looseness in the system was modelled according to Fig. 4, where all the bolts at the bearing pedestal P3, in node 109 (n109), were loosened. The bolts’ axes were coincident with the vertical direction of the general coordinates and a clearance $\:{c}_{L}=1\:\mu\:m$ exists within the surface, allowing a restricted displacement in the vertical direction. As a result, there is a stiffness reduction in the rotor support, and a nonlinear behaviour is observed.

In this model, the effects of the loosened bolts are strictly applied to P3 node’s coordinates and only in the vertical direction. The stiffness values in the horizontal direction are not affected, thereby remaining the same as the tightened model.

In the general equation of motion (Eq. 5), the $\:\boldsymbol{K}$ matrix is modified depending on the vertical displacement at the loosened location. The static equilibrium position is at * y=0*, with the rotor resting on the surface. Assuming an elastic behaviour in the supports, three possibilities were identified regarding the vertical position of the rotor during operation as described below and represented in Eq. 11.

i. Displacement is negative, the surface is compressed, and the vertical stiffness $\:{k}_{vv}$ is the same as in the tightened pedestal; therefore, no additional forces regarding looseness arise.
ii. Displacement is between zero and the given clearance, motion in the vertical direction is free, and no restrictions are imposed by the support. There is no stiffness contribution to the system in the vertical direction due to the loose pedestal, and no forces arise.
iii. Displacement is higher than the defined clearance, the bolts are in traction, and the stiffness value at this location is the same as in the tightened pedestal.

$$\:y<0\:\:\:\:\:\:\:\:\:\:\:\:\:\:\left\{\begin{array}{c}{k}_{vv}=\:{k}_{vv}\:\\\:{f}_{Lv}=0\:\:\:\:\:\:\:\:\:\:\:\:\:\:\:\end{array}\right.$$

$$\:0\le\:y\le\:{c}_{L}\:\:\:\:\:\:\left\{\begin{array}{c}{k}_{vv}=0\:\:\\\:{f}_{Lv}=0\end{array}\right.$$

11 $$\:y>{c}_{L}\:\:\:\:\:\:\:\:\:\:\:\:\:\:\left\{\begin{array}{c}{k}_{vv}={k}_{vv}\:\\\:{f}_{Lv}={c}_{L}\cdot\:{k}_{vv}\end{array}\right.$$

The support stiffness contribution will act only after the clearance is reached. Therefore, the elastic force $\:{\boldsymbol{K}}_{\boldsymbol{s}}({q}_{i}-{q}_{c})$ is added to the equation of motion (Eq. 5); where $\:{\boldsymbol{K}}_{\boldsymbol{s}}$ is the matrix containing the loosened support contribution to stiffness, $\:{q}_{i}$ is the displacement of the rotor at the instant *i*, and $\:{q}_{c}$ is the vector containing the clearance at the loosened support with all its other components equal to zero.

Being $\:{\boldsymbol{K}}_{0}$ the stiffness matrix of the system without the loosened support contribution, the equation of motion can be written as Eq. 12, where $\:{{\boldsymbol{K}}_{\boldsymbol{s}}q}_{\boldsymbol{c}}$ is the force $\:{F}_{L}$ that arises due to the loosened pedestal, of which the only component different to zero is at the pedestal node in the vertical direction, $\:{f}_{Lv}$.

12 $$\:\boldsymbol{M}\ddot{q}+\left({\Omega\:}\boldsymbol{G}+\boldsymbol{C}\right)\dot{q}+{(\boldsymbol{K}}_{0}{+\boldsymbol{K}}_{\boldsymbol{s}})q={{\boldsymbol{K}}_{\boldsymbol{s}}q}_{\boldsymbol{c}}$$

### Partial rub

According to Goldman and Muszynska^40^, the impact between rotational and stationary parts in mechanical systems is mainly addressed under three different approaches. In the first, the impact is considered to be instantaneous and elastic. In the second, the restitution coefficient is zero, thus the impact is non-elastic and followed by a sliding stage, thereby altering the rotor speed during its occurrence. Finally, in the third approach, there is a consideration that the impact generates modifications to the dynamic characteristics of the rotor stiffness and damping.

In this study, the rotor rub effects are included through the modification of the stiffness matrix, neglecting the effects on the damping values.

**Fig. 5:**
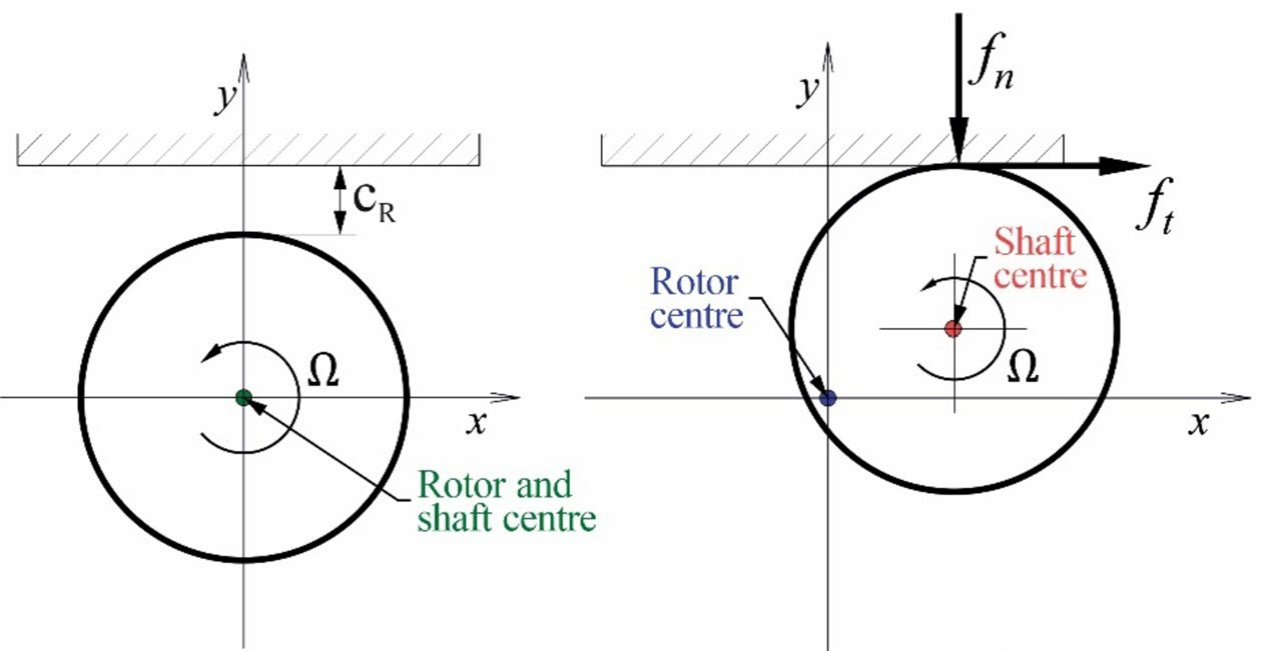
Partial rub diagram.

The fault is simulated as shown in Fig. 5, where a partial rub exists due to contact between the rotor and a rigid static surface, parallel to the horizontal plane, located in the positive vertical direction. The clearance between both elements during the equilibrium position is $\:{c}_{R}=3.0\mu\:m$, at node 39 (n39).

When impact occurs, the rotor is subjected to two forces, $\:{f}_{n}$ in the negative vertical direction and its respective friction force $\:{f}_{t}={\mu\:}_{k}\cdot\:{f}_{n}$ in the negative horizontal direction, with $\:{\mu\:}_{k}=0.3$ the kinetic friction coefficient.

Similar to the mechanical looseness model described in the previous section, two possible conditions are given due to the rotor’s vertical position at the n39, as explained below.

i. Displacement is lower than the defined clearance in the vertical direction, free motion is observed in the rotor, and no contact occurs. In this case, the equation of motion remains as Eq. 5.
ii. Displacement is higher than the defined clearance in the vertical direction, and contact exists between the rotor and the stator. The rotor stiffness increases in both horizontal and vertical directions, due to the stator effect. A high value of stator stiffness in the vertical direction is adopted for modelling purposes $\:{k}_{s}=100MN/m$. Then, the equation of motion is developed following the same considerations as the looseness model and adding the friction force acting in the horizontal direction, resulting in Eq. 13. In the expression, the matrix $\:{\boldsymbol{K}}_{\boldsymbol{s}\boldsymbol{t}}$ has only two elements different to zero, both at n39, $\:{k}_{uu}={\mu\:}_{k}\cdot\:{k}_{s}$ and $\:{k}_{vv}={k}_{s}$. The vector containing the forces produced by rub, $\:{F}_{R}$, also has only two components different to zero, both at n39 displacement directions $\:{f}_{u}={\mu\:}_{k}\cdot\:{k}_{s}\cdot\:{c}_{R}$ and $\:{f}_{v}={k}_{s}\cdot\:{c}_{R}$.

13 $$\:\boldsymbol{M}\ddot{\boldsymbol{q}}+\left({\Omega\:}\boldsymbol{G}+\boldsymbol{C}\right)\dot{\boldsymbol{q}}+\left(\boldsymbol{K}{+\boldsymbol{K}}_{\boldsymbol{s}\boldsymbol{t}}\right)\boldsymbol{q}={F}_{R}$$

## Combined defects and rotor responses

In practice, rotating machines are sometimes subjected to multiple defects occurring simultaneously at different levels of severity. Any faulty condition, if not corrected in a timely manner, could lead to other faults, irreversible damage to the equipment, thereby creating other knock-on effects (including significant downtime, loss of income, penalties, safety, environment, etc.)

Using the available experimental data measured in the modelled rig as a reference, the defects described in the previous sections are introduced into the FE model. The initial baseline condition from the experiments is subject to residual unbalance and residual misalignment. This is also true for the remaining studied cases, i.e. bent shaft, looseness in the pedestal and rotor rub. Therefore, the dynamic responses from the studied machine are estimated in a combined form, aiming to replicate real-life scenarios of manufacturing or assembly imperfections. Further details of the simulated scenarios are summarised in Table 5.

**Table 5:** FE simulated scenarios.

1	Scenario	Defects included in FE model	Operational speed (s)
	Baseline	Residual unbalance and residual misalignment	* 1800rpm* * 2400rpm*
2	Bent shaft	Shaft bow, residual unbalance and residual misalignment	* 1800rpm* * 2400rpm*
3	Looseness	Looseness in pedestal, residual unbalance and residual misalignment	* 1800rpm* * 2400rpm*
4	Rub	Partial rotor rub, residual unbalance and residual misalignment	* 1800rpm* * 2400rpm*
5	Combined bent shaft and rub	Shaft bow, partial rotor rub, residual unbalance and residual misalignment	* 1800rpm*

The residual unbalance fault was considered to occur at D3 location, i.e. n128, with an eccentricity $\:\epsilon\:=0.03\:mm$ and an offset angle $\:\beta\:$, randomly selected within the range of $\:0-2{\uppi\:}\:rad$. In addition, to account for the residual misalignment effects, the rotor responses are estimated using a vertical offset of $\:\varDelta\:y=-0.1mm$ and a horizontal offset of $\:\varDelta\:x=0.8mm$. These values were determined iteratively to reproduce the characteristic spectral components associated with both faults, namely the $\:1\times\:$ and $\:2\times\:$ harmonic responses typically observed in the FFT.

**Fig. 6:**
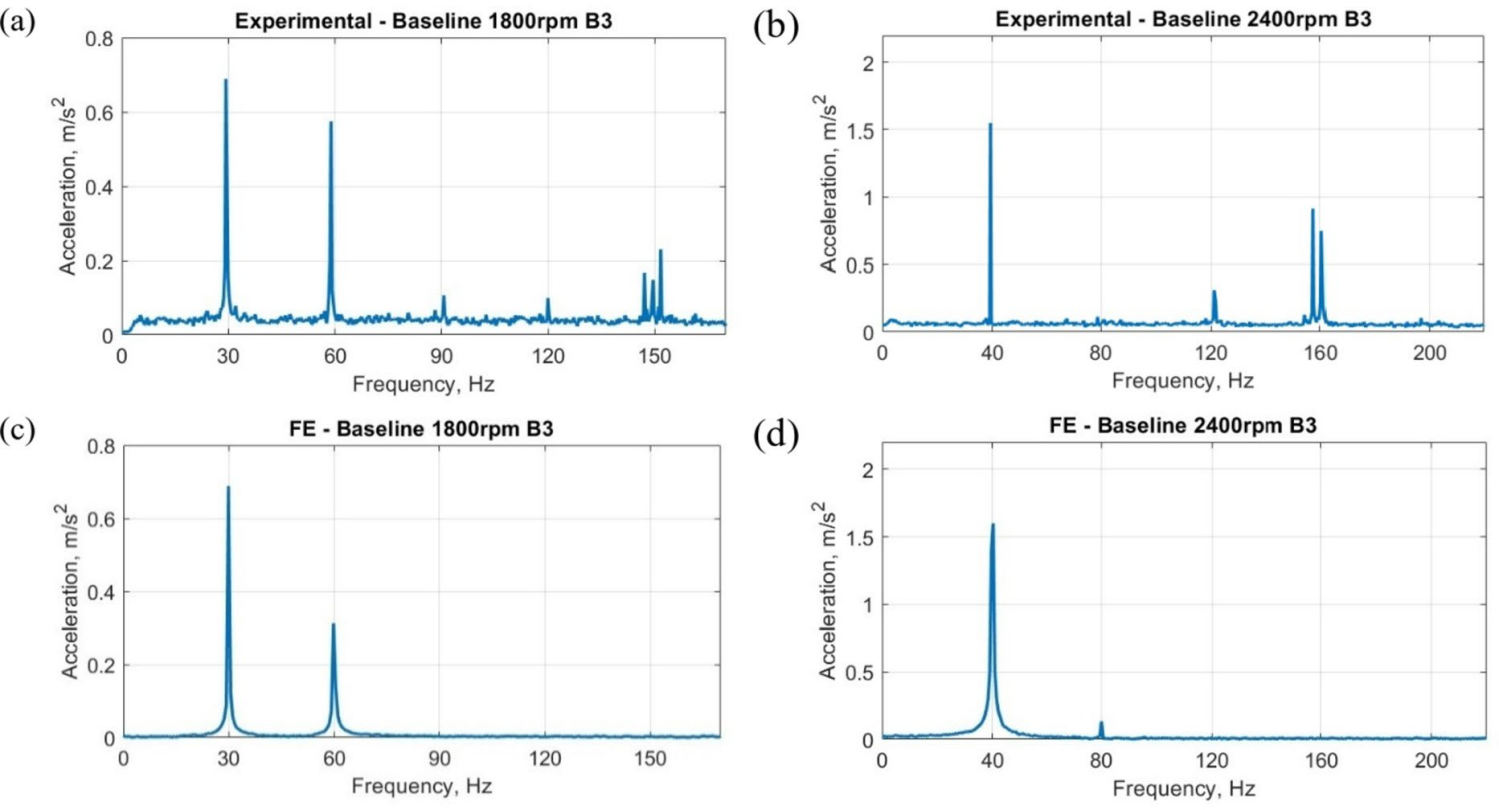
Spectra of acceleration response for rotor baseline condition at B3. (**a**) Experimentally measured, * 1800rpm*. (**b**) Experimentally measured,$\:\:2400rpm$. (**c**) Estimated in FE model, * 1800rpm*. (**d**) Estimated in FE model, * 2400rpm*.

In Fig. 6, the acceleration spectra calculated from the experimentally measured signals, along with those generated from the FE model, are presented at both operational speeds, namely * 1800rpm* and * 2400rpm*.

As expected, the FE-generated spectra contain only the first and second harmonics of the fundamental frequency, reflecting the influence of unbalance forces acting at $\:1\times\:$, and misalignment forces acting at both $\:1\times\:$, and $\:2\times\:$. However, at * 2400rpm*, the $\:1\times\:$, component becomes dominant, indicating the increased contribution of the speed-dependent unbalance and misalignment forces to such component relative to the misalignment-only induced $\:2\times\:$ response.

**Fig. 7:**
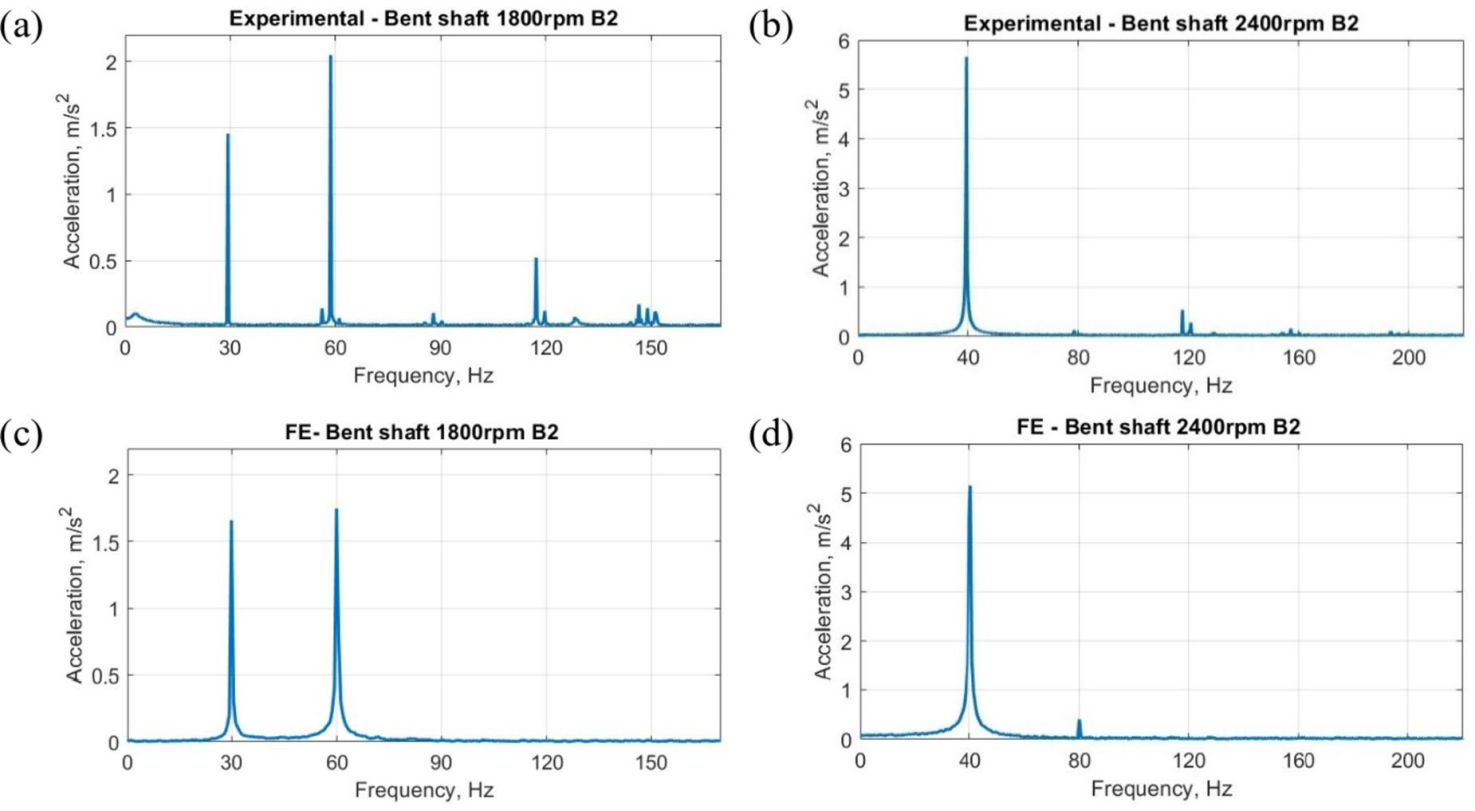
Spectra of acceleration response for bent shaft measured at B2. (**a**) Experimental, * 1800rpm*. (**b**) Experimental,$\:\:2400rpm$. (**c**) FE model, * 1800rpm*. (**d**) FE model, * 2400rpm*.

In Fig. 7, the acceleration spectra obtained at B2 when the bent rotor is running at * 1800rpm* and * 2400rpm* are shown for both experimentally and numerically acquired data. The occurrence of bow generates eccentricity along the shaft, and the observed responses appear as unbalance but at higher values. The effects on the faulty rotor under increased operational speed are evidenced here.

**Fig. 8:**
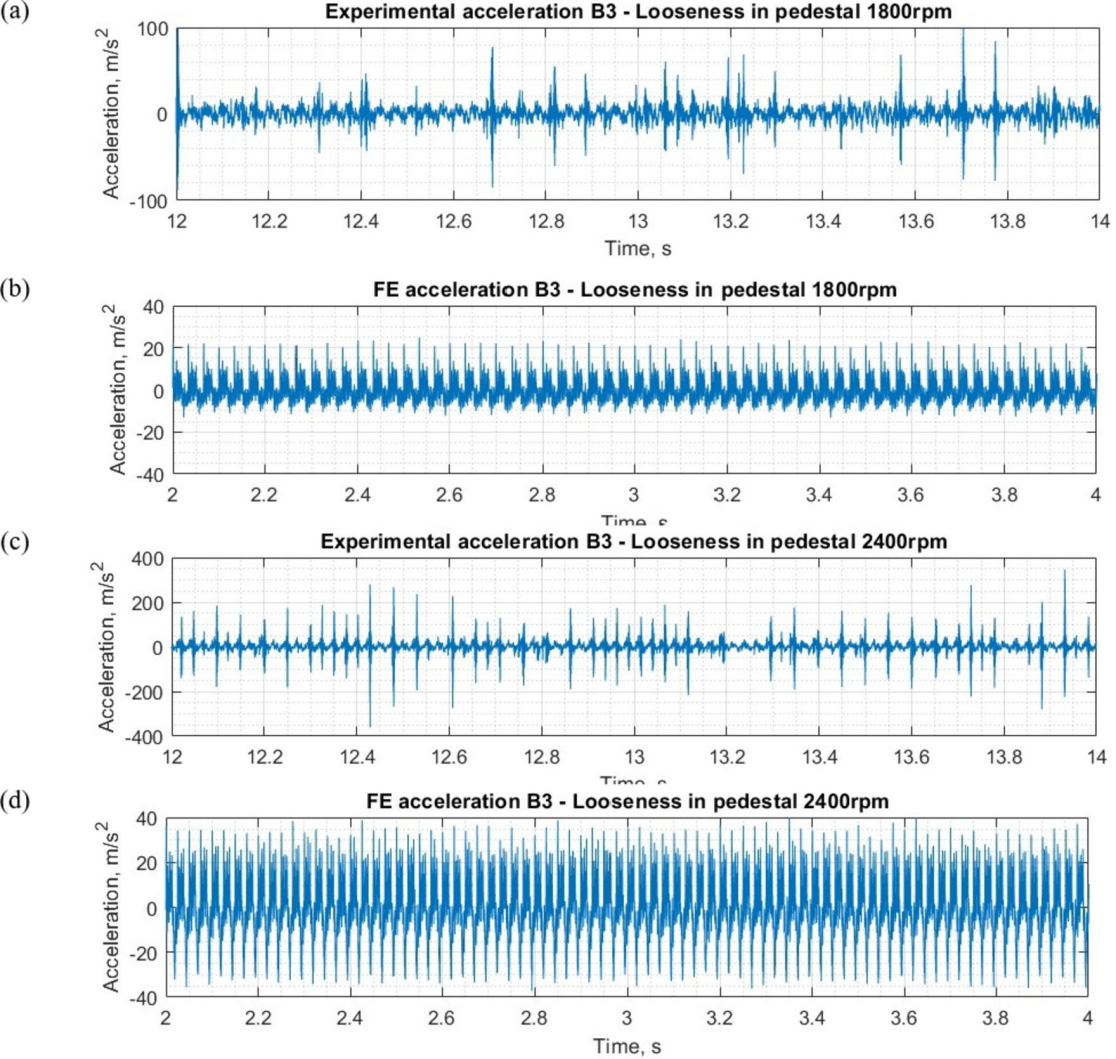
Acceleration rotor responses in time domain at B3 location with looseness in P3. (**a**)Experimental - * 1800rpm*. (**b**)FE - * 1800rpm*. (**c**)Experimental - * 2400rpm*. (**d**) FE - * 2400rpm*.

When the rotor is subjected to pedestal looseness, the vibration responses obtained from both the experimental measurements (Fig. 8(a) and (c)) and the corresponding FE simulations (Fig. 8(b) and (d)) provide clear and consistent evidence of the governing fault mechanism across the two investigated rotational speeds. The transient impact events observed in the datasets arise from intermittent loss of structural support at pedestal P3, which induces abrupt local variations in the system’s stiffness and the nonlinear behaviour characteristic of a loosened support.

**Fig. 9:**
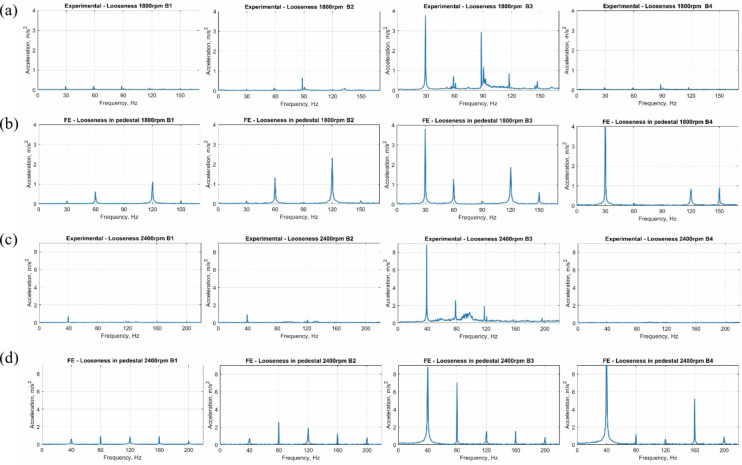
Spectra of acceleration response for looseness in pedestal P3, measured at B1 – B4. (**a**) Experimental, * 1800rpm*. (**b**) FE model, * 1800rpm*. (**c**) Experimental,$\:\:2400rpm$. (**d**) FE model, * 2400rpm*.

In the acceleration spectra, harmonics occurring at multiples of the rotational speed were observed at all 4 bearing locations. As expected, B3 shows higher amplitude components, confirming the location of the loosened pedestal. This is consistent with the observations in the experimental signals (Fig. 9).

In the acceleration spectra, harmonics occurring at multiples of the rotational speed were observed at all four bearing locations. As expected, bearing B3 exhibits the highest amplitude responses, confirming its association with the loosened pedestal and aligning with the experimental observations shown in Fig. induced behaviour at both inv9(a) and (c). However, the numerical results in Fig. 9(b) and (d) reveal a notable deviation from the experimental behaviour, where the 1$\:\times\:$ component displays elevated amplitude at bearing B4, whereas the $\:2\times\:$ and higher order harmonics consistently remain dominant at B3 across all studied scenarios. This discrepancy between experimental and simulated responses at B4 can be attributed to the modelling assumptions used to incorporate residual unbalance and misalignment. These assumptions can amplify location-specific effects, particularly when impulsive forces arise due to the intermittent stiffness changes at the loosened pedestal.

**Fig. 10:**
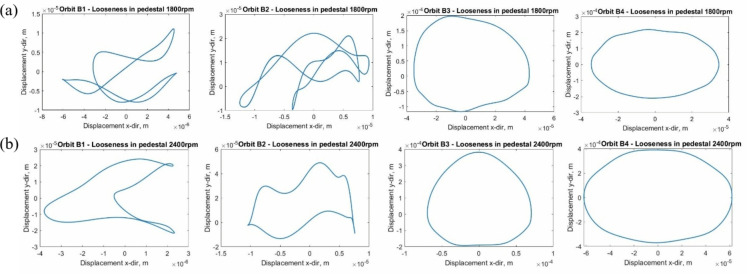
Orbit plots for looseness, measured at B1, B2, B3 and B4. (**a**) FE model, * 1800rpm*. (**b**) FE model, * 2400rpm*.

Although the frequency spectra and overall system responses clearly indicate the presence of looseness, the orbit plots at the loosened location, B3, display features that resemble primarily unbalance and misalignment-induced behaviour at both investigated speeds (Fig. 10). This can be attributed to the comparatively high amplitude of the fundamental rotational speed component, which becomes particularly influential at B3 and B4, producing orbits characteristic of unbalance. In contrast, the orbits observed at B1 and B2 remain more typical of misalignment-related responses. Further comparison with experimental orbits is not feasible at this stage, as only acceleration datasets from the former experiments are currently available.

**Fig. 11:**
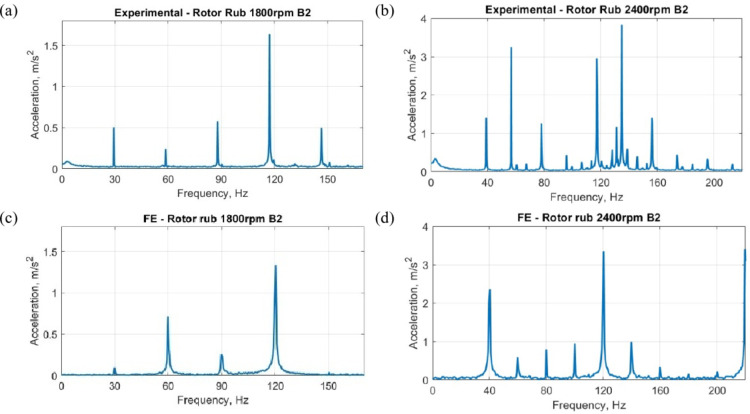
Spectra of acceleration response for rotor rub measured at B2. (**a**) Experimental, * 1800rpm*. (**b**) Experimental,$\:\:2400rpm$. (**c**) FE model, * 1800rpm*. (**d**) FE model, * 2400rpm*.

In the rub scenarios, although the acceleration spectra show higher amplitudes at the B2 location at both speeds (Fig. 11), the displacement analysis provides additional insight into the system’s behaviour. At * 1800rpm*, the simulated displacement responses indicate that the highest RMS (root mean square) value occurs at the B4 location. At * 2400rpm*, however, the B2 location shows the largest RMS value. The RMS values obtained from the simulated rub signals are presented in Table 6.

**Table 6:** RMS values from displacement signals in time domain generated in FE model, rub scenario.

Location	$\:1800\boldsymbol{r}\boldsymbol{p}\boldsymbol{m}$		$\:2400\boldsymbol{r}\boldsymbol{p}\boldsymbol{m}$	
	$\:\boldsymbol{x}-\boldsymbol{d}\boldsymbol{i}\boldsymbol{r}$	$\:\boldsymbol{y}-\boldsymbol{d}\boldsymbol{i}\boldsymbol{r}$	$\:\boldsymbol{x}-\boldsymbol{d}\boldsymbol{i}\boldsymbol{r}$	$\:\boldsymbol{y}-\boldsymbol{d}\boldsymbol{i}\boldsymbol{r}$
**B1**	$\:3.3148\times\:{10}^{-3}$	$\:10.5801\times\:{10}^{-3}$	$\:2.7899\times\:{10}^{-3}$	$\:30.0141\times\:{10}^{-3}$
**B2**	$\:5.0681\times\:{10}^{-3}$	$\:5.0761\times\:{10}^{-3}$	$\:9.1892\times\:{10}^{-3}$	$\:52.6831\times\:{10}^{-3}$
**B3**	$\:3.9119\times\:{10}^{-3}$	$\:15.9916\times\:{10}^{-3}$	$\:6.6153\times\:{10}^{-3}$	$\:28.9150\times\:{10}^{-3}$
**B4**	$\:2.8287\times\:{10}^{-3}$	$\:17.4157\times\:{10}^{-3}$	$\:0.1851\times\:{10}^{-3}$	$\:6.9799\times\:{10}^{-3}$

**Fig. 12:**
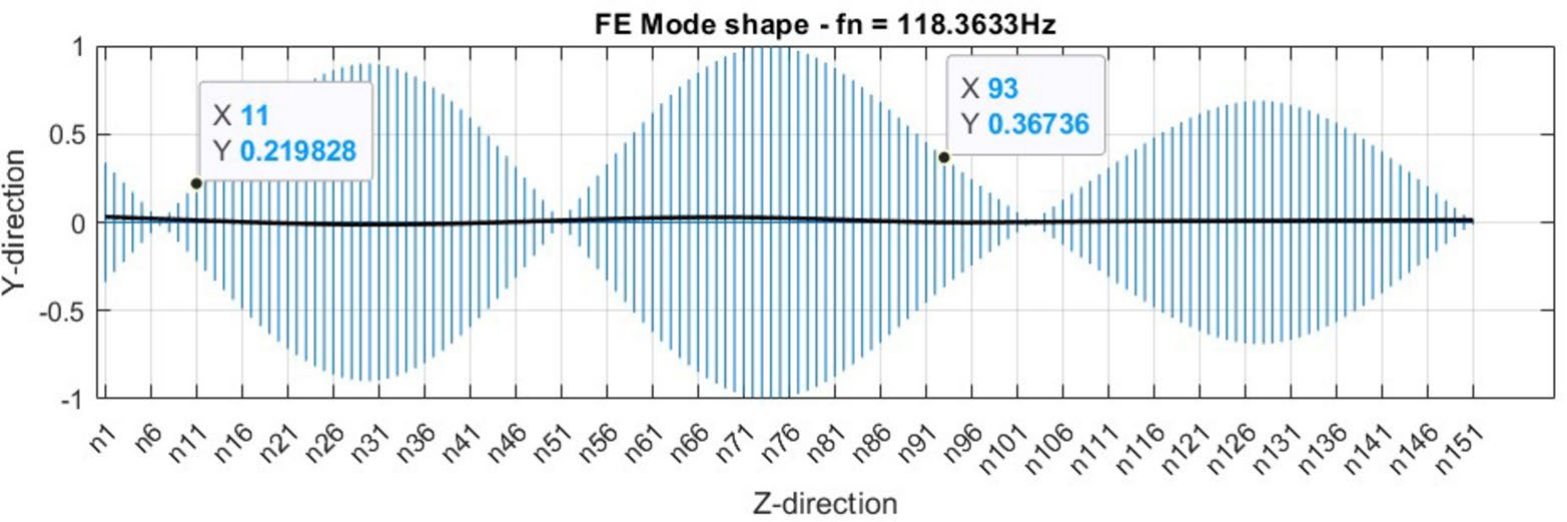
FE simulated mode shape, for natural frequency * 118.3633Hz*.

As shown in Fig. 11, higher harmonics of the fundamental rotational frequency are present at both investigated speeds. At * 2400rpm*, additional spectral components appear at multiples of half the fundamental frequency, as illustrated in Figs. 9(b) and 9(c). A prominent * 120Hz* component is consistently observed at both speeds; previous studies have identified this frequency as being close to one of the system’s natural frequencies. This observation is corroborated by the finite element model, in which the third natural frequency is calculated as * 118.3633Hz*. The corresponding mode shape (Fig. 12) indicates that excitation near this frequency results in a significantly larger vibration response at B2 (n93) compared with B1 (11), explaining the observed amplification at B2 despite the contact occurring closer to B1.

**Fig. 13:**
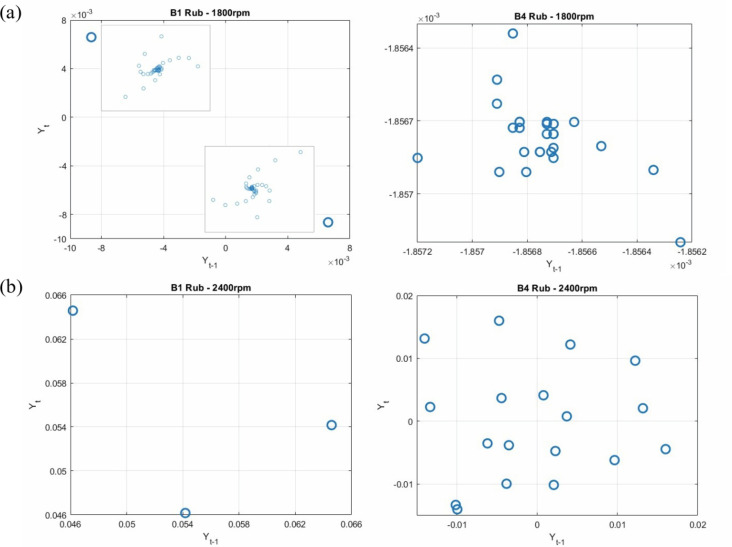
Poincaré map for vertical displacement at B1 and B4, Rub FE simulation. (**a**) * 1800rpm*. (**b**) * 2400rpm*.

**Fig. 14:**
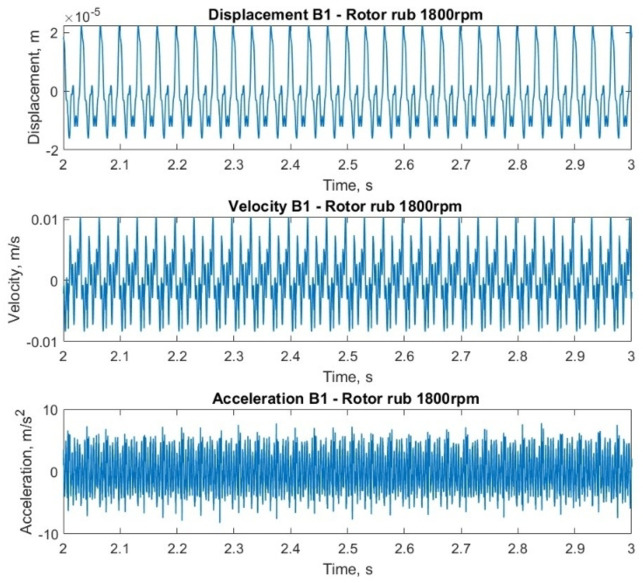
Rotor responses estimated in time domain in FE model with rub, operating at * 1800rpm* for B1 location.

**Fig. 15:**
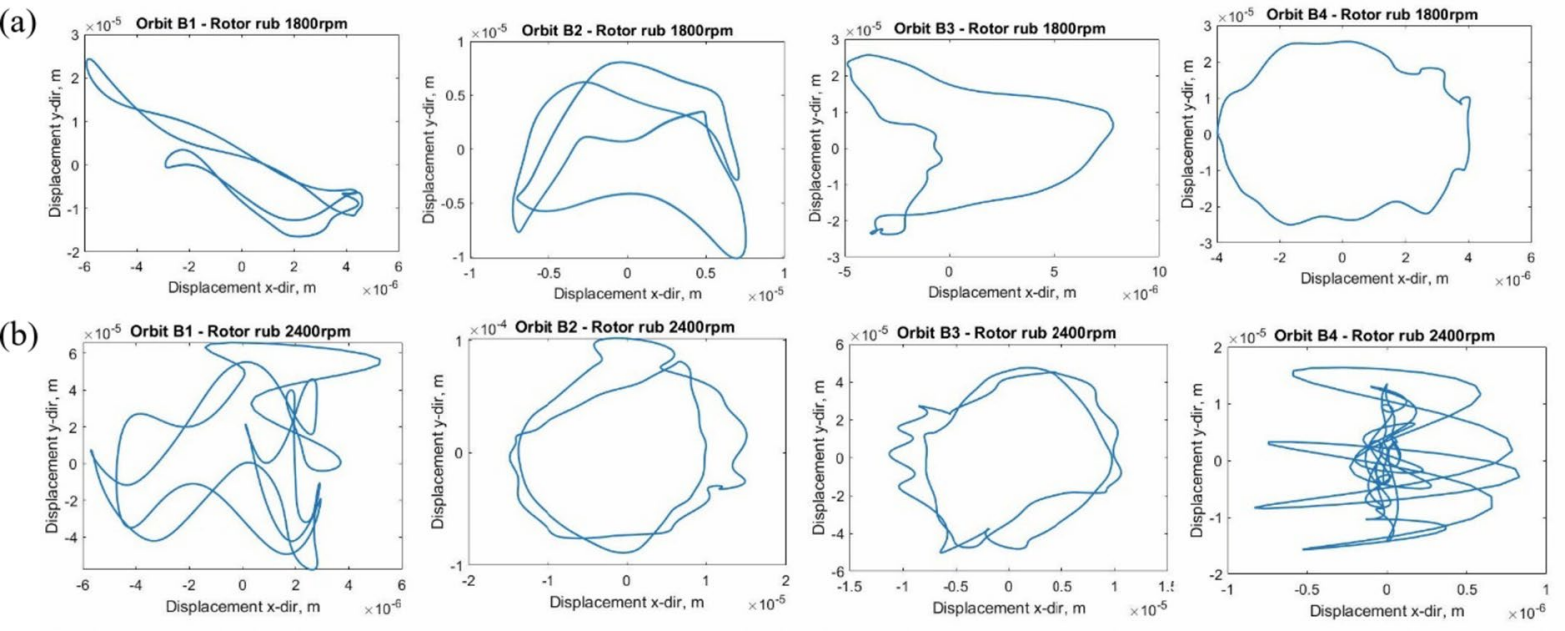
Orbit plots for rotor rub, measured at B1, B2, B3 and B4. (**a**) FE model, * 1800rpm*. (**b**) FE model, * 2400rpm*.

The time domain responses presented in Fig. 14 reveal the characteristics associated with the occurrence of rub, consistent with the expected transient contact behaviour of rotor–stator interaction. The corresponding orbit plots in Fig. 15 further highlight the influence of fault mechanisms on the system’s lateral responses. The trajectories observed at bearings B2 and B3 align most closely with the patterns typically induced by misalignment and residual unbalance^27,41^, reflecting the dominance of fundamental and low order harmonic components under these conditions. The orbits measured at B1 for both operating speeds, as well as at B4 at * 2400rpm*, exhibit distinct nonlinear behaviour. The Poincaré maps in Fig. 13 show evidence of chaotic motion at B1 and B4 at * 1800rpm*, and again at B4 at * 2400rpm*. This response is consistent with rub-induced nonlinearities, which arise from intermittent variations in rotor–stator contact stiffness. In contrast, the Poincaré map at B1 at * 2400rpm* indicates a quasi-periodic rotor response. This trend accords with established observations in the literature, wherein increased rotational speed promotes transient variations in system stiffness, shifts natural frequencies, and facilitates the onset of chaotic dynamics in rub dominated operations.

**Fig. 16:**
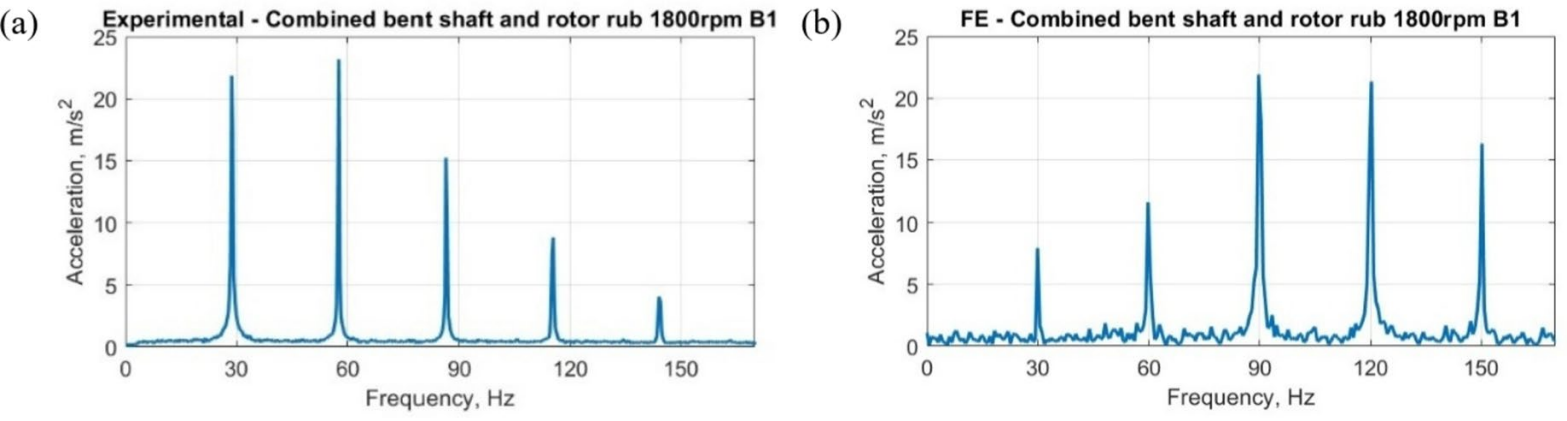
Spectra of acceleration response for combined bent shaft and rotor rub, measured at B1. (**a**)Experimental, * 1800rpm*. (**b**) FE model, * 1800rpm*.

The most complex scenario combines the effects of rotor rub, bent shaft, residual unbalance and residual misalignment at * 1800rpm*. The obtained acceleration spectrum at B1 in the FE model in Fig. 16(b) shows an opposite trend to the experimental measurements in Fig. 16(a), with harmonic components of the fundamental increasing in amplitude at higher frequencies. This discrepancy is attributable to the nonlinear interaction between the phase angles associated with the bent shaft and the residual unbalance, whose combined influence becomes more prominent in the presence of impulsive contact forces.

**Fig. 17:**
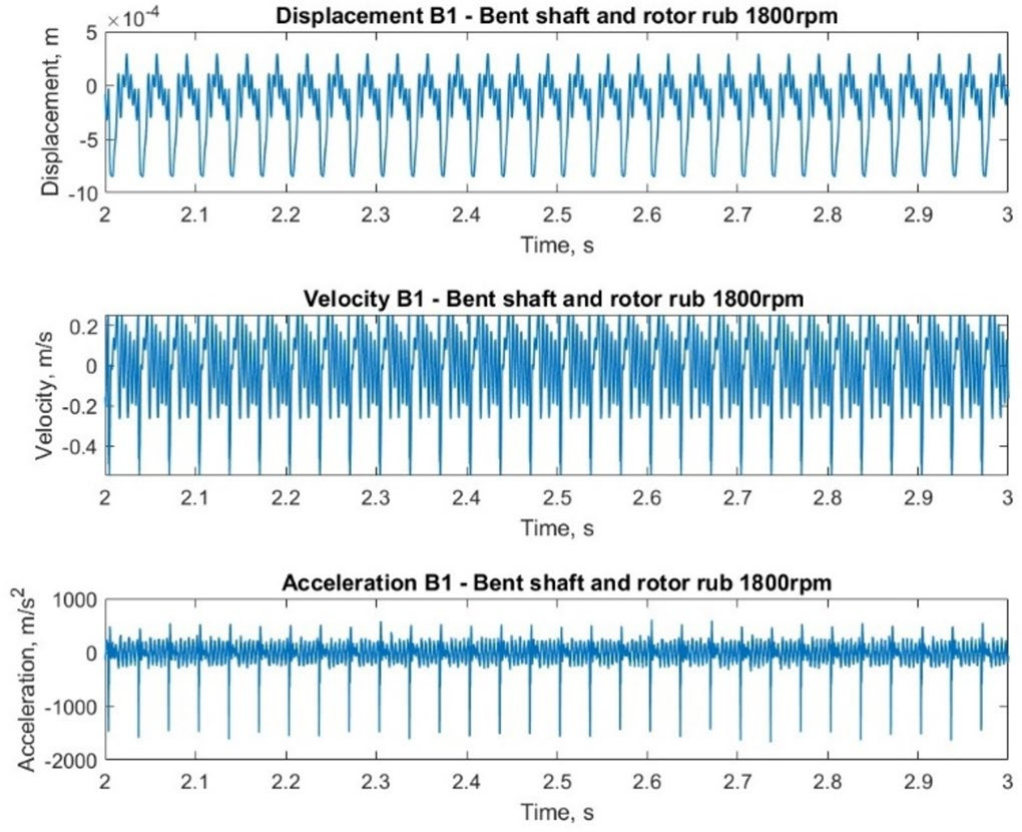
Rotor responses estimated in the time domain in the FE model with bent shaft and rub, operating at * 1800rpm* for the B1 location.

**Fig. 18:**
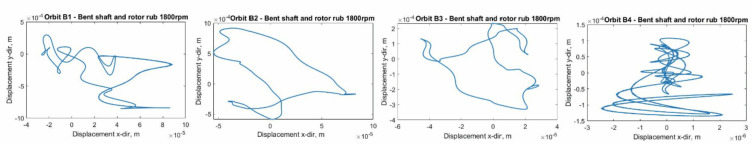
Orbit plots for bent shaft and rotor rub, measured at B1, B2, B3 and B4. FE model, $\:1800\:rpm$.

The impulsive nature of the dynamic responses due to the rotor-stator contact is observed in the time domain plots (Fig. 17). The orbit plots in Fig. 18 show a similar behaviour as observed in Scenario 4, where only misalignment, unbalance and rub defects were included in the simulation. While the shape of the orbits as well as the presence of super-harmonics in the spectra suggest a dominance of the rub over the bent shaft, the eccentricity in the rotor produced by the bent shaft has a clear effect on the amplitude of the responses.

A summary of the key parameters extracted from the acceleration spectra of both the experimental measurements and the finite element simulations is presented in Table 7 for * 1800rpm* and Table 8 for * 2400rpm*. The parameters include the amplitudes of the 1× to 5× harmonic orders, as well as the corresponding spectral energy at these frequencies.

Overall, the simulated dynamic responses show good agreement with the experimental results. Nevertheless, differences are observed in the extracted parameters, which are expected and can be attributed to the simplifying assumptions and approximations adopted during the model development. In particular, certain effects such as detailed bearing dynamics were not explicitly modelled and may influence the measured response. Despite these limitations, the FE model captures the main dynamic features of the system and remains a valuable tool for investigating the interaction of simultaneous rotor faults, as commonly encountered in real industrial applications.

**Table 7:** Summary of amplitudes (A) and spectrum energy (SE) for experimental and FE scenarios at * 1800rpm*, up to 5×.

		1×		2×		3×		4×		5×	
		E	FE	E	FE	E	FE	E	FE	E	FE
**Baseline** **(B3)**	**A (m/s** ^**2**^ **)**	0.69	0.09	0.20	0.31	-	-	-	-	-	-
	**SE (± 10** ***df*** **)**	74.09	38.75	109.24	54.83	-	-	-	-	-	-
**Bow** **(B2)**	**A (m/s** ^**2**^ **)**	1.46	0.21	2.05	1.74	-	-	-	-	-	-
	**SE (± 10** ***df*** **)**	55.89	94.73	173.77	299.65	-	-	-	-	-	-
**Looseness** **(B3)**	**A (m/s** ^**2**^ **)**	3.77	0.48	0.43	1.30	0.25	0.16	0.19	1.90	0.15	0.62
	**SE (± 10** ***df*** **)**	335.88	218.14	218.69	214.98	770.36	70.69	452.61	797.86	426.23	249.00
**Rub** **(B2)**	**A (m/s** ^**2**^ **)**	0.50	0.01	0.24	0.71	0.58	0.23	1.64	1.34	0.50	0.05
	**SE (± 10** ***df*** **)**	30.55	9.55	51.70	121.56	131.49	102.84	485.84	560.79	246.09	44.53
**Rub + Bow** **(B1)**	**A (m/s** ^**2**^ **)**	21.85	0.82	23.16	9.57	15.24	14.21	8.82	22.16	4.05	18.49
	**SE (± 10** ***df*** **)**	2405.80	769.74	3533.53	2234.59	4273.11	6198.97	4049.73	9571.08	2757.54	7881.42

**Table 8:** Summary of amplitudes (A) and spectrum energy (SE) for experimental and FE scenarios at * 2400rpm*, up to 5×.

		1×		2×		3×		4×		5×	
		E	FE	E	FE	E	E	FE	E	FE	E
**Baseline** **(B3)**	**A (m/s** ^**2**^ **)**	1.55	1.60	0.11	0.13	-	-	-	-	-	-
	**SE (± 10** ***df*** **)**	112.22	257.59	104.98	25.54	-	-	-	-	-	-
**Bow** **(B2)**	**A (m/s** ^**2**^ **)**	5.66	5.14	0.12	0.38	-	-	-	-	-	-
	**SE (± 10** ***df*** **)**	652.71	835.10	65.89	74.22	-	-	-	-	-	-
**Looseness** **(B3)**	**A (m/s** ^**2**^ **)**	8.82	8.73	2.57	6.99	1.91	1.48	0.41	1.51	0.54	0.87
	**SE (± 10** ***df*** **)**	695.26	1414.02	1158.13	806.44	1587.04	610.56	710.95	523.36	1009.16	622.70
**Rub** **(B2)**	**A (m/s** ^**2**^ **)**	1.41	2.29	1.25	0.82	2.95	3.31	1.41	0.35	0.32	0.19
	**SE (± 10** ***df*** **)**	127.22	370.00	286.43	137.41	1182.27	1400.73	888.73	177.65	376.22	259.78

## Conclusions

This study has presented an investigation into the dynamic behaviour of a multi–fault rotor–bearing assembly, using a finite element (FE) framework based on Timoshenko beam theory. Five common rotor-related defects, namely unbalance, misalignment, shaft bow, pedestal looseness, and partial rub, were examined with special focus on combined occurrence scenarios to reflect realistic industrial conditions. By integrating experimentally derived modal parameters and measured vibration responses, the developed FE model was calibrated and validated to accurately replicate the characteristic features of the rig’s behaviour under multiple operational states.

The results demonstrate that when a dominant fault acts in combination with residual unbalance and misalignment, the FE model is capable of reproducing the key spectral and time-domain features observed experimentally, at both investigated speeds. These findings confirm the model’s suitability for capturing the principal mechanisms governing typical single fault conditions. More importantly, the study identifies distinct fault-specific signatures, such as speed-dependent harmonic patterns, orbit characteristics, and transient impacts, that persist even when faults act simultaneously. These signature features represent valuable indicators for fault discrimination and have direct relevance for condition monitoring, feature engineering, and the development of physics-informed failure management tools.

However, the research also highlights the modelling challenges that arise when two or more severe defects interact nonlinearly. In highly coupled conditions, such as simultaneous bent shaft and rub faults, the FE predicted acceleration spectra deviate from the experimentally observed trends. These discrepancies stem from intricate interactions between fault induced forces, phase relationships, and impulsive contact phenomena—interactions that are difficult to capture fully within a simplified FE representation. Such findings underscore the importance of continued refinement of physics-based models to better represent nonlinearities associated with intermittent contact, support flexibility, and multi-fault coupling.

Overall, this work provides new insight into the dynamic behaviour of rotating machinery subjected to combined fault conditions. It establishes a strong foundation for future developments in physics-guided feature extraction and intelligent fault diagnosis. Planned future work will extend this modelling framework to incorporate fault severity quantification, fault location sensitivity, and advanced parameter estimation techniques. These improvements will support the development of robust, high-fidelity models capable of guiding predictive maintenance strategies and enhancing reliability across a wide range of industrial systems. While the focus of the current study is on rotor-related combined faults in flexibly supported rotating machine models, the relevance of understanding the contributions of the bearing support systems and other rotor configurations (e.g., overhung rotors) to fault dynamics cannot be overemphasised. Therefore, the consideration of more transient scenarios, different rotor configurations, and bearing defects by future studies would enhance the robustness of the proposed finite element modelling approach.

## Data Availability

The theoretical and experimental dynamic characteristics data sets that support the findings of this study are available from the University of Manchester’s Research Data Repository. Except for the publicly available data sets that form part of previously published preliminary investigations, access is only available to current staff members and students. The data are, however, available upon request and with the permission of the University of Manchester Research Management and Export Control teams.
